# The Learning Curve for Hand-Assisted Laparoscopic Total Gastrectomy in Gastric Cancer Patients

**DOI:** 10.3390/jcm11226841

**Published:** 2022-11-19

**Authors:** Philippa Seika, Matthias Biebl, Jonas Raakow, Dino Kröll, Candan Çetinkaya-Hosgör, Peter Thuss-Patience, Max Magnus Maurer, Eva Maria Dobrindt, Johann Pratschke, Christian Denecke

**Affiliations:** 1 Chirurgische Klinik, Campus Charité Mitte/Campus Virchow-Klinikum, Charité Universitätsmedizin Berlin, 13353 Berlin, Germany; 2 Department of Surgery, Ordensklinikum Linz, 4020 Linz, Austria; 3 Medizinische Klinik mit Schwerpunkt Hämatologie, Onkologie und Tumorimmunologie, Campus Charité Mitte/Campus Virchow-Klinikum, Charité Universitätsmedizin Berlin, 13353 Berlin, Germany; 4 BIH Charité Clinician Scientist Program, Berlin Institute of Health, 13353 Berlin, Germany

**Keywords:** gastric surgery, learning curve, CUSUM, minimally invasive gastrectomy, gastric cancer

## Abstract

(1) Background: Hand-assisted laparoscopic total gastrectomy (LTG) for patients with gastric cancer (GC) has been established as the standard surgical treatment at our center. This study aims to quantify the learning curve for surgeons performing minimally invasive total gastrectomy at a high-volume single center. (2) Methods: One hundred and eighteen consecutive patients who underwent minimally invasive total gastrectomy between January 2014 and December 2020 at a single high-volume center were included and reviewed retrospectively. Risk-adjusted cumulative sum analysis (RA-CUSUM) was used to monitor the surgical outcomes for patients with different risks of postoperative mortality using varying-coefficient logistic regression models. Patients were ordered by the sequential number of the procedure performed and divided into two groups according to the degree of surgeon proficiency as determined by RA-CUSUM analysis (group A: 45; group B: 73 patients). Age, gender, body mass index (BMI), tumor location, pathology, and comorbidities were compared while primary endpoints comprised surgical parameters, postoperative course, and survival outcomes. (3) Results: Forty-four cases were required for the completion of the learning curve. During this time, the mean operating time decreased. Hand-assisted laparoscopic total gastrectomy performed after a learning curve was associated with a shorter median operating time (OT) (360 min vs. 289 min, <0.001), and a reduced length of stay (A = 18.0 vs. B = 14.0 days) (*p* = 0.154), while there was a trend toward less major complications (Clavien–Dindo (CD) 3–5 within 90 days (12 (26.67%) vs. 10 (13.70%) *p* = 0.079). Our results showed no difference in anastomotic leakage between the two groups (group A vs. group B, 3 (6.67%) vs. 4 (5.48%) *p* = 0.99). Similarly, 30-day (0 (0%) vs. 1 (1.7%), *p* = 0.365) and 90-day mortality (1 (2.08%) vs. 2 (3.39%), *p* = 0.684) were comparable. Following multivariate analysis, the level of surgical proficiency was not a significant prognostic factor for overall survival. (4) Conclusions: A minimum of 44 cases are required for experienced laparoscopic surgeons to achieve technical competence for performing LTG. While operation time decreased after completion of the learning curve, quality criteria such as achievement of R0 resection, anastomotic leakage, and perioperative mortality remained unaltered. Of note, the level of surgical training showed no significant impact on the 2 year OS or DFS.

## 1. Introduction

While hand-assisted laparoscopic total gastrectomy (LTG) is considered technically feasible for the treatment of early gastric cancer [1,2,3], its applications have been extended from early gastric cancer to advanced disease including the increased treatment of patients with T3 and T4 tumors [4,5,6]. With growing expertise and proficiency, a reduction in blood loss and surgery time with LTG has been achieved while maintaining a comparable oncological outcome. Structured surgical training programs play an important role in ensuring that a standard of oncological care is maintained throughout the teaching process. Nevertheless, such training programs can only be implemented when surgical techniques are established. Quantitative assessment of the learning curve during the adoption of a new procedure is necessary to guide the development and implementation of training programs for fellows. Furthermore, training programs can be tailored accordingly in terms of how long the fellowship should be, how many fellows can be accommodated by a given institution, and what supplementary training can be offered to adjunct operative training.

However, concerns about the technical difficulty and oncological safety of LTG in advanced gastric cancer remain. LTG and transhiatal extended LTG (teLTG) are considered technically challenging operations requiring considerable training. On the other hand, the operation time or length of hospital stay play an increasing role, and thus, patient safety, surgical quality, and economic parameters must all be considered when analyzing a surgical learning curve. While some studies have assessed the number of cases required to achieve proficiency in LTG, most have concentrated on cases from Asian hospitals with limited literature from the Western world. Several reports have suggested regional differences in the incidence of gastric cancer. Differences in stage-stratified surgical outcome and survival are also reported between Asian and Western populations with gastric cancer [7]. Therefore, we analyzed our experience after more than 100 procedures in a Western population. The cumulative sum (CUSUM) analysis is a method that has been applied in the medical literature to quantify the feasibility and oncological safety of the procedure and as a reliable method to assess the quality of surgical performance based on outcome. While useful, the CUSUM method treats all patients identically and fails to consider differences in clinical presentations and individual risk factors. As a result, a risk-adjusted cumulative sum failure (RA-CUSUM) method was performed.

This study aimed to define the institutional learning curve associated with the minimally invasive total gastrectomy for experienced laparoscopic surgeons in a Western population to establish a reliable and consistent proficiency.

## 2. Patients and Methods

This study examined the learning curve of the institutional adoption of a novel surgical technique in a university hospital. The surgical department comprises two campuses and is accredited as a tertiary referral center for gastroesophageal surgery by the German society for general and visceral surgery (DGAV). In our institutional training program, the trainee joins the laparoscopic gastrectomy program after prior surgical experience in bariatric surgery and laparoscopic upper gastrointestinal (upper GI) surgery, followed by open total gastrectomy. They then proceed to work under the supervision of an experienced surgeon who has performed at least fifty laparoscopic gastrectomies as well as minimally invasive endoscopic, robotic, and, in this instance, also colorectal procedures. All operations in this study were performed by two senior consultants with substantial expertise in open total gastrectomy, minimally invasive esophageal resections, and laparoscopic colon resections. All procedures were assisted by one attending and one upper GI fellow as per standard procedure in our hospital.

### 2.1. Inclusion and Exclusion Criteria

**Figure 1:**
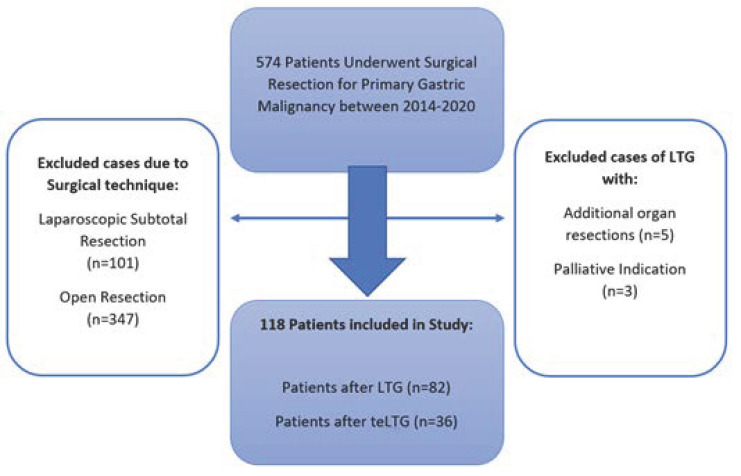
Study exclusion criteria flow chart.

A total of 574 patients underwent surgical resection for primary gastric malignancy between January 2014 and December 2020 at our center (Figure 1). Of these patients, all patients who underwent open procedures or laparoscopic distal or subtotal gastrectomy were excluded from this study. Furthermore, analysis was performed by including only LTG in the absence of concomitant procedures. The remaining 118 patients were included.

### 2.2. Data Collection

Demographic details, perioperative data such as age, sex, BMI, operating time (OT), duration of postoperative hospital stay, pTNM staging and postoperative complications including anastomotic leakage (AL), 30- and 90-day morbidity and mortality as well as follow up data (overall survival (OS) and disease-free survival (DFS) were reviewed retrospectively from a prospectively maintained database. Perioperative complications were classified according to the Clavien–Dindo classification [8] and complications of grade 3 to 5 were considered major postoperative complications. Anastomotic leakage (AL) was defined as a defect of the intestinal wall at the anastomotic site, leading to communication between the intra- and extraluminal compartments. All cases reported were verified endoscopically or via X-ray or computed tomography (CT)-imaging with an orally administered contrast agent.

### 2.3. Surgical Procedure

**Figure 2:**
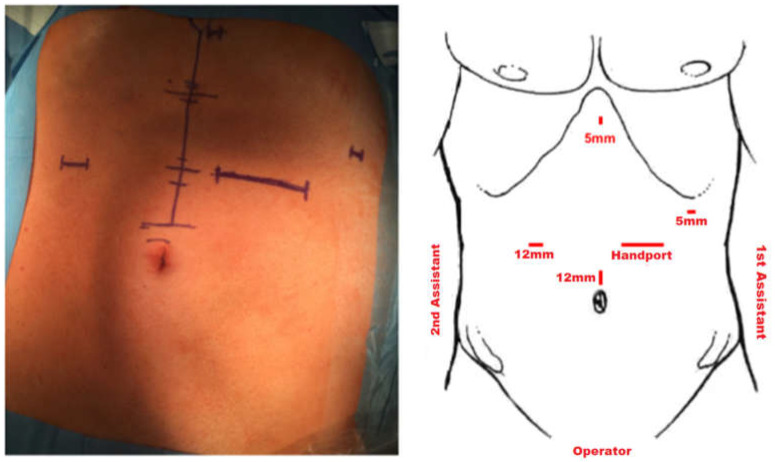
Trocar placement.

Hand-assisted laparoscopic surgery has been the standard procedure for early and locally advanced gastric cancer at our institution since 2014 and is performed using a hand-assisted technique. Hand-assisted laparoscopic gastrectomy has the advantages of simpler operation, shorter operative time, and does not compromise the postoperative recovery period. The patient is positioned in a supine position. Initially, open entry into the abdominal cavity is via a muscle-sparing transrectal approach in the left mid-abdomen and placement of the hand port (GelPort, Applied Medical, Rancho Santa Margarita, CA, USA), which is then either used for a hand-assisted approach or laparoscopically via placement of a 12 mm trocar through the gel membrane. Entry into the abdominal cavity is followed by exploration of the situs. Subsequently, further trocars are placed as shown in Figure 2. With the right hand of the operator inserted through the hand port, the operator then lifts the stomach and omentum and uses an ultrasound knife to separate the splenogastric ligament and transect the short gastric vessels. Following oncological resection and D2-lymphadenectomy as the standard procedure in Germany, transection of the duodenum and esophagus is performed with a linear stapler (EchelonFlex powered; Johnson & Johnson Medical GmbH, Ethicon, USA, and EndoGIA Covidien, respectively) and reconstruction is performed by Roux-Y esophagojejunostomy. In the case of transhiatal resection, the distal esophageal dissection and local lymphadenectomy (LAD) is accomplished through opening the diaphragmatic hiatus up to 7 cm anteriorly. Specifically, preparation of the alimentary limb and jejunojejunostomy is conducted in an open fashion while the esophagojejunostomy is performed hand-assisted as a 25 mm circular anastomosis (powered Echelon, Johnson & Johnson Medical GmbH, Ethicon, Raritan, NJ, USA) using the Orvil-anvil system (Medtronic).

Intraoperative pathologic evaluation is performed in all patients by frozen section examination to ensure tumor-free resection margins. In the case of tumor infiltration, further resection is performed until tumor-free resection margins are confirmed by frozen section examination.

The integrity of the anastomosis was verified by intraoperative endoscopy and air leak testing. Before closing the abdominal cavity, a perianastomotic drain was placed. Operating time (OT) reported is defined by the time from incision to completion of wound closure.

### 2.4. Multimodal Therapy

Multimodal therapy can nowadays be considered standard in patients with preoperative lymph node positive disease or a tumor stage >T2 and is routinely performed in our center according to a tumor board decision based on the German S3 guideline. As such, neo-adjuvant chemotherapy is followed by postoperative chemotherapy. All tumor stages (TNM) given in this study refer to postoperative histological classifications unless specified otherwise.

### 2.5. Perioperative Management

All patients were admitted to a surgical intensive care unit postoperatively. Following the commencement of oral fluid intake on the first postoperative day and early physical mobilization from the first postoperative day, gradual enteral food intake was commenced on the third POD, providing peristalsis was unimpaired. Perianastomotic drains were removed if the discharge was less than 150 mL/24 h.

All resected specimens were routinely histologically evaluated for TNM classification and evaluation of resection margins concerning the presence of tumor cells.

### 2.6. Defining the Learning Curve of Hand Assisted Laparoscopic Total Gastrectomy for Gastric Cancer

Surgical procedures require perioperative surveillance and monitoring in order to detect any changes in the process. Despite the expertise of the surgeons, mastering a new technique requires practice, particularly considering that LTG is a challenging operation. Control charts are commonly used to monitor for changes in a process by distinguishing between special causes and common causes of variation and therefore present an ideal method for quantifying the learning curve of LTG. The cumulative sum (CUSUM) was employed in order to detect a change in the process and the respective cause in terms of operative time. Moreover, we used RA-CUSUM to establish an estimate for the process change point in LTG in terms of perioperative outcome (90 day morbidity and mortality). This allowed us to consider further influencing parameters affecting surgical outcome such as complexity of the procedure (transhiatal extended), stage of disease, and patient characteristics (age, gender, BMI, ASA score, pre-existing conditions, and prior abdominal operations). Multivariable logistic regression was performed to guide the selection of significant parameters for perioperative outcome, which were then employed in risk adjustment. RA-CUSUM guided the cut of point, and the two groups were further analyzed comparatively.

### 2.7. Statistical Analysis

First, the learning curve analysis was performed according to the cumulative sum CUSUM method to identify the relationship between single parameters (in this case, operating time) and the sequential number of the procedure performed. To account for possible patient-related factors influencing perioperative outcome (90 day morbidity and mortality), a risk-adjusted CUSUM (RA-CUSUM) was performed. Patients were then divided into groups according to the cutoff point of the RA-CUSUM graph. Finally, the two groups were analyzed comparatively.

The patient demographics in group A and group B were stratified by histologic type, pathologic T status, N status, and postoperative adjuvant chemotherapy. A comparison of these datasets was performed by the Student’s *t*-test or analysis of variance (ANOVA) for continuous variables, and x^2^ was chosen for the categorical data. Multivariate analysis was performed using the Cox proportional hazards model. A *p*-value below 0.05 was considered significant. Survival analysis was performed using the Kaplan–Meier method.

For the analysis of the learning curves, the cumulative sum technique (CUSUM) was used. CUSUM is a sequential analysis technique designed to detect changes in a parameter of the probability distribution [9]. For CUSUM analysis of OT, patients were ordered chronologically according to the date of surgery. The simple CUSUM series was defined for every case as CUSUMn = ∑ (Xn − X0) + CUSUMn-1, where Xn represents the individual measurement, and X0 is a predetermined reference level. X0 was set as the mean for all cases in the first analysis. In our cohort, the mean OT was 317 min. The control limit was set at +/−2.5.

Risk adjusted CUSUM calculations were conducted as described in [9] using dynamic probability control limits [10] for the assessment of signals. Learning was defined in terms of a reduction in the perioperative 90-day morbidity (Clavien Dindo 3–5) within 90-days of LTG and subsequently weighted by the individual risk. Patient risks were calculated via a bias-reduced generalized linear regression model [11]. The data were included in the model when the *p*-value was less than 0.05. Predictors for outcome included in our risk-adjustment model were age, ASA score, and OT validated in an independent cohort. The risk scores derived from the regression model were substituted for X0 for each successive case in the CUSUM equation. On the RA-CUSUM chart, a negative slope represents improved results compared with those predicted, whereas deterioration in performance results in a positive slope. The RA-CUSUM chart was set to reset once the control limit had been reached.

Statistical analysis was performed using R (version 3.2.4., R Foundation for Statistical Computing, Vienna, Austria) and CUSUM calculations were conducted via R package ‘cusum’.

## 3. Results

### 3.1. Defining the Learning Curve

The learning curve was defined both in terms of operating time (OT) by cumulative sum analysis (CUSUM), and risk-adjusted cumulative sum analysis (RA-CUSUM). Specifically, RA-CUSUM was employed to calculate the number of cases required until the proficiency achieved a reduction in the perioperative 90-day morbidity and mortality (Clavien Dindo 3–5). Learning curve analysis allowed two distinct groups to be identified: group A was considered the training group, and group B the competent group.

### 3.2. Cumulative Sum of Differences (CUSUM) Method

**Figure 3:**
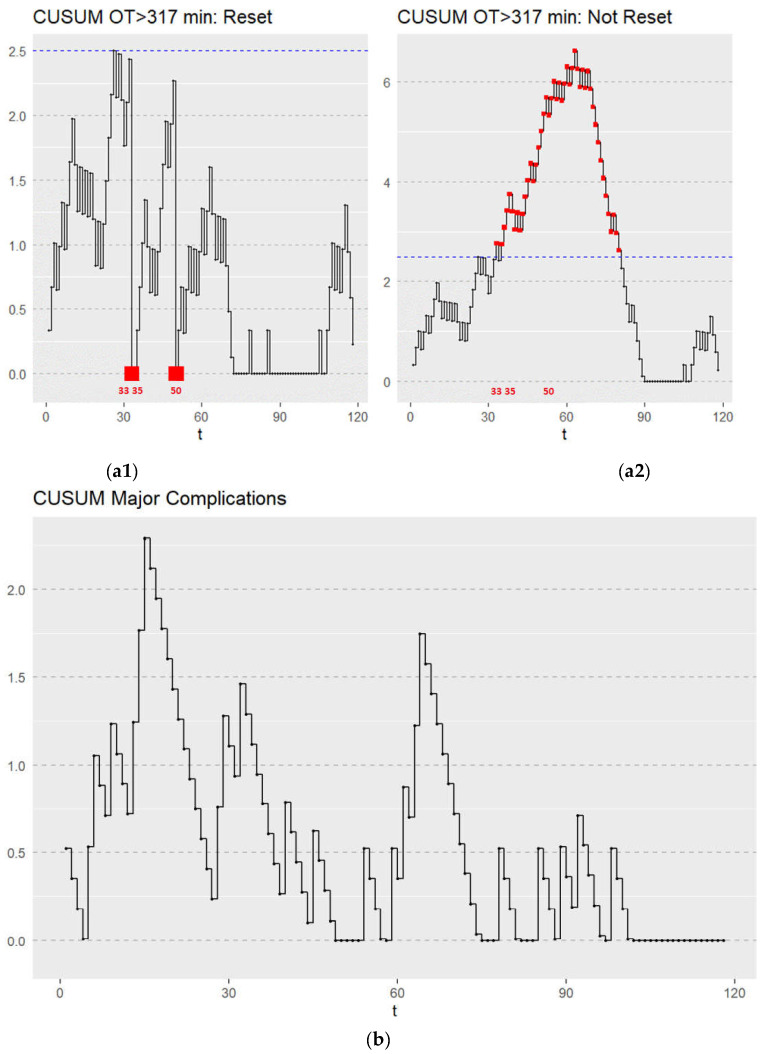
(**a1**) CUSUM graph depicting the surgical learning curve for LTG in terms of OT. Failure was defined as an OT > 317 min. CUSUM OT > 317 min triggered a signal at 33, 35, and 50 patients. All CUSUM runs were initiated with C0 = 0 and reset Ct to zero after every alarm. The upper control limit is depicted by the blue dotted line. Points which triggered a signal are marked with a red point. (**a2**) CUSUM graph depicting the surgical learning curve for LTG in terms of OT. Failure was defined as an OT > 317 min. Initially increasing values of Ct leveled off after the fiftieth OT case and then decreased with increased learning. t—sequential hand-assisted laparoscopic gastrectomy performed. The upper control limit is depicted by the blue dotted line. Points which triggered a signal are marked with a red point. (**b**) CUSUM graph depicting the surgical learning curve for LTG in terms of major complications (CD 3–5), t—sequential hand-assisted laparoscopic gastrectomy performed.

The learning curve was first assessed by the CUSUM method. There are a number of varied models that are commonly applied in learning curve analysis [9,10]. Generally, a number of increasing values of the CUSUM values Ci indicate a higher-than-expected rate of the adverse event, while decreasing values indicate a better than expected performance relative to what is expected [9,10]. When the control limit is reached, the chart (C = 0) may not be reset. Figure 3(a1,a2) shows the CUSUM graph depicting the surgical learning curves for LTG in terms of operating time using two commonly reported CUSUM applications [9,10]. The target value was defined as the mean operating time in our cohort of <317 min. Regarding the OT, the learning process was completed after 50 cases. The CUSUM graphs (Figure 3(a1,a2)) for OT >317 min signal at 33, 35, and 50 patients indicate that the upper control limits (set at 2.5) were reached in these cases. As seen in Figure 3(a2), a plateau was reached after the fiftieth case with a subsequent decline in the CUSUM chart. This method is useful in providing visual aids, however, due to the lack of well-defined decision rules and performance metrics, we focus on the resetting model [10] shown in Figure 3(a1) in the remainder of this article.

The CUSUM graph for major complications (Figure 3b) showed a stable process throughout the learning curve with neither the upper nor lower control limits (set at 2.5) being reached (Figure 3b). A rate of major complications was maintained throughout the learning process at an acceptable rate with no deviations. The limit of acceptable major complications was set at 0.186 (18.6%) according to the mean of our cohort. To summarize the results from our non-risk adjusted CUSUM charts, the institution reached a proficiency in terms of OT after 50 cases, while proficiency concerning major complications was consistent throughout the learning process.

### 3.3. Risk-Adjusted Cumulative Sum Analysis (RA-CUSUM)

In order to address the complexity of this type of surgery, which is influenced by a multitude of additional factors (i.e., patient demographics), the learning curve was further evaluated by the RA-CUSUM method, as previously described. To estimate the risk of 90-day morbidity and mortality for LTG, we used a logistic regression model based on all LTG from 2014 to 2020 including the following covariates: age, OT, and ASA score. The model’s discriminative ability was assessed using the goodness of fit (deviance = 776.206 and AICc = 802.7). The pseudo-R2 was 0.511. and model mis-classification rate was 5.08%. Signals were assessed by using dynamic probability control limits [10].

**Figure 4:**
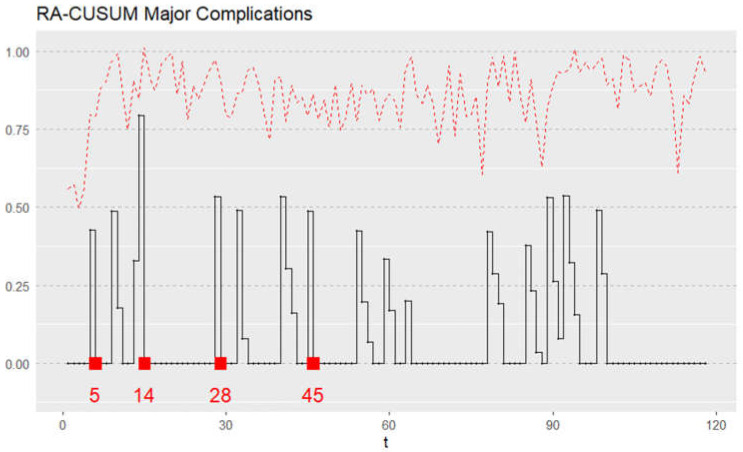
Risk-adjusted cumulative sum (RA-CUSUM) model for surgical failure considered in terms of either major complication (Clavien Dindo, grade  ≥ 3) or 90-day mortality. t—sequential hand-assisted laparoscopic gastrectomy performed.

Each patient’s risk-adjusted ratio was plotted sequentially using the risk-adjusted CUSUM graph depicted in Figure 4. The RA-CUSUM graph signaled an alarm in cases 5, 14, 28, and 45. No further signals occurred after the forty-fifth case, suggesting that at this point, a stable process was achieved. Therefore, we can conclude that group A represents the initial learning phase curve up to the forty-fifth case, while all cases beyond this point, comprise the competent phase (group B).

### 3.4. Patient Demographics

The cohort consisted of 118 patients with a mean age of 63.8 (29.0–89.0) years, of which 82 were male, and 36 were female (Table 1). An American Society of Anesthesiologists (ASA) score of ≥3 was seen in 59.0% of patients. The mean body mass index (BMI) was 26.31 (15.0–37.2) kg/m^2^.

The indication for gastrectomy was adenocarcinoma in 112 patients including 20 (16.9%) patients with a signet ring cell carcinoma (SRCC) (Table 2). Histopathological tumor characteristics included an even distribution of intestinal (*n* = 56 (47.5%)) and diffuse (*n* = 45 (38.1%)) histological subtypes across the cohort. Two patients received gastrectomy due to a neuroendocrine tumor (NET), two patients presented with a mixed adenoneuroendocrine carcinoma (MANEC), one patient presented with a gastric metastasis of malignant melanoma, and one patient received prophylactic gastrectomy due to a CDH-1 gene defect. A transhiatal extended gastrectomy was performed in 36 (30.5%) patients.

The demographic data are shown in Table 1. Some differences were seen in terms of tumor location, with significantly more tumors located in the antrum in group B (*n* = 11 (24.44%) vs. *n* = 31 (42.47%), *p* = 0.047) and more tumors located in the corpus in group A (*n* = 26 (57.78%) vs. *n* = 27 (36.99%), *p* = 0.027).

No differences were seen in terms of operation type (total gastrectomy (*n* = 33 (73.33%) vs. *n* = 47 (64.38%), *p* = 0.31) and teLTG (*n* = 12 (26.67%) vs. *n* = 26 (35.62%), *p* = 0.312), respectively. The duration of the operation varied significantly between the two groups (360 min vs. 289 min, <0.001). Similarly, the LN yield was significantly greater in group B (group A vs. group B, 27.83 vs. 34.51, <0.001) while both groups met the oncological safety criteria as defined by the German guidelines (LN yield of *n* > 25). No significant difference was seen in the ratio of affected LN (0.122 vs. 0.072 (*p* = 0.24)). There was a trend toward a shorter postoperative hospital stay in group B; this, however, was not significant (A = 18.0 vs. B = 14.0 days) (*p* = 0.154).

### 3.5. Histopathological Data

The primary malignancy did not vary significantly between the groups. G1–G3 adenocarcinoma was the most common indication for gastrectomy (*n* = 43 (95.56%) vs. *n* = 68 (93.15%) *p* = 0.58). In histological evaluation, a non-significant trend was seen toward more patients with SRCC in group B (*n* = 10 (22.22%) vs. *n* = 30 (41.10%), *p* = 0.58). The distribution of all histopathological subtypes did not vary between the groups.

The study included 24 patients with a T3 or T4 stage in group A and 35 patients in group B (*n* = 35 (68.89%) vs. *n* = 35 (47.95%), *p* = 0.57). (Table 2). Importantly, advanced tumor stages (T3/T4) as well as nodal stages and grade of differentiation were equally distributed. One patient in each group A and three patients in group B showed a positive resection margin upon postoperative histopathological examination (*n* = 1 (2.22%) vs. *n* = 3 (4.11%), *p* > 0.99).

### 3.6. Postoperative Outcomes

Major and minor postoperative complications defined as Clavien Dindo 1–5 occurred in 56 patients (47.46%), with 22 (48.89%) complications in group A and 34 (46.58%) complications in group B (*p* = 0.964). Major complications (Clavien Dindo ≥ 3) accounted for 12 cases in group A (26.67%) and 10 cases (13.70%) in group B (*p* = 0.079). The details of the complications are listed in Table 3. In total, there were seven cases of anastomotic leakage (4 grade IIIa and 1 grade IIIb) amongst the entire cohort with no differences between the groups (group A vs. group B, *n* = 3 (6.67%) vs. *n* = 4 (5.48%), *p ≥* 0.99) Within the first 90 postoperative days, one patient in group A and two patients in group B died (1 (2.08%) vs. 2 (3.39%), *p* = 0.684). No differences in 1-year survival rate (A = 93.3% vs. B = 96.9%) or 2-year survival rate (A = 90.6% vs. B = 92.5%) were seen between the two groups.

Next, risk factors for major complications were evaluated. Upon univariate analysis, major complications were affected by age (0.460 (0.189–1.120), *p* = 0.041), ASA score (2.550 (1.065–6.107) *p* = 0.036), and an OT > 360 min (3.954 (1.578–9.911), *p* = 0.003). However, only age (5.101 (1.787–14.562), *p* = 0.002) and OT > 360 min (4.494 (1.484–13.614), *p* = 0.008) were identified as significant risk factors following multivariate analysis (Table 4).

### 3.7. Survival

**Figure 5:**
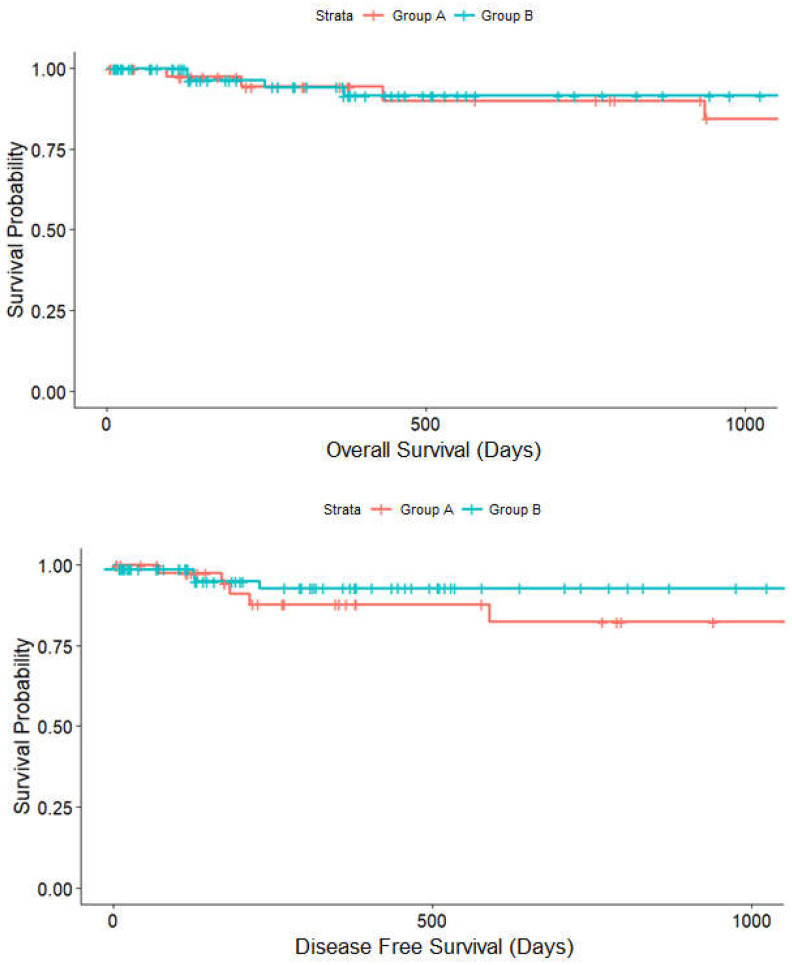
Kaplan–Meier analysis on the effect of surgical training group A (red line) and group B (blue line) on disease-free survival (*p* = 0.111) and overall survival (*p* = 0.787).

The median follow-up period was 726.12 (±102.439) days in group A and 477.00 (±50.131) days in group B. There was a trend toward higher 2-year DFS rates in group B (83%) than in group A (74%) (*p* = 0.111) (Figure 5) while OS did not vary (group A vs. group B, 93.3% vs. 96.9%, *p* = 0.111). At last follow-up, tumor recurrence had occurred in 27 patients, while 11 patients died in total.

Upon Cox regression analysis, the presence of SRCC (*p* = 0,049 HR = 3.019 (1.003–9.088)) or an R1 resection status (*p* = 0.003 HR = 12.815 (2.350–69.871)) were significant factors negatively associated with DFS (Table 5). In contrast, overall survival was significantly affected by R1 resection status (*p* = 0,024 HR = 19.103 (1.473–247.692)) and a prolonged OT of >360 min (*p* = 0.050 HR = 0.234 (0.055–1.000)).

## 4. Discussion

Since the first reported case of LTG in 1994 [12], hand-assisted laparoscopic surgery for early gastric cancer has been established as the standard procedure. Many studies have demonstrated the benefits of minimally invasive surgery including reduced postoperative complications, reduced postoperative pain, and a shorter recovery period [13,14,15]. Advances in laparoscopic gastrectomy enabling an improved perioperative outcome while maintaining oncological safety have led to increased adoption of the procedure in more complex cases. However, the procedure is commonly associated with a steep learning curve [1,16,17,18], with differences in outcome reported between trainee and experienced surgeons. Oncological and surgical safety must be measured and maintained throughout fellowship programs for trainee surgeons. A quantitative evaluation of the learning curve in upper gastrointestinal surgery may inform the design and implementation of such training programs.

Quality control in surgery is an increasing field of interest as the consistent provision of optimal results is required. Although the learning curves for some major laparoscopic procedures including distal gastrectomy have been established using the CUSUM and RA-CUSUM methods [16], this approach has not been applied to LTG in the Western population [16]. The application of these tools may be beneficial to guide how, and in what time frame, an operative procedure must be taught in order to maximize patient safety.

It has been previously reported that an experience of approximately 50–60 cases of LTG is required to achieve proficiency. However, the reported number of cases required is often unspecific, with a large range varying from 20–100 cases [17,19]. The learning curve is often reported by comparing surgical outcomes using crude survival or perioperative data without considering variabilities in the case-mix. Jung et al. reported a learning phase followed by an intermediate phase of 50 cases, respectively, with completion of the learning curve after 100 cases using a simple moving average method [19]. However, this method was based on the mean operative time and mean estimated blood loss (EBL), which does not allow for precise identification of a changepoint. Similarly, Jin et al. reported on the learning curve completion after 60 cases using CUSUM analysis. While the authors hypothesized that patient selection influenced the learning curve considerably, the analysis did not use risk adjustment or account for variations in the case-mix [1].

In this study, the minimum surgical failure was observed by the forty-fifth case, which is shorter than most learning curves described in the literature. One reason for this may be that the training for hand-assisted laparoscopic oncological upper gastrointestinal surgery at our institute includes a thorough training in bariatric and reflux surgery prior to advancing to hand-assisted laparoscopic total gastrectomy. Thus, substantial laparoscopic skills were acquired by surgeons prior to the beginning of the learning curve. Furthermore, the anastomotic technique partly varies between institutions, and therefore our learning curve, a hand-assisted 25 mm circular esophagojejunostomy, may be an easier technique to master than others, comparatively shortening our learning curve.

However, LTG was not yet an established primary treatment option at the beginning of this study. In 2014, the first year included in this study, three cases of LTG were performed. The learning curve may have been negatively impacted during the early phase of learning due to the low initial rate of cases, which may have prolonged the learning period. As such, the rate of operations is a factor in the duration of the learning curve [18,19]. The risk adjustment for the complexity of patients performed in our study, however, may not allow for a direct comparison of studies without a risk adjustment.

There are two options for establishing a new technique: either learning by doing and modifying the technique as experience is gained, or a laparoscopically trained surgeon adopts a standardized technique from an already experienced center, which may lead to a steeper learning curve. The latter procedure was conducted at our institution as laparoscopic surgeons were experienced in LDG and bariatric surgery and adopted further details from standardized techniques. This prior experience may expedite the process of learning LTG.

Based on our experience, we conclude that establishing a standardized surgical training protocol may help in shortening the LTG learning curve. Such a standardization of surgical learning may include fellowship programs. Such programs should include the development of basic laparoscopic skills and the understanding of advanced surgical techniques (i.e., lymph node dissection or intracorporeal stapling techniques prior to performing LTG. Furthermore, using a laparoscopic training device as implemented in our clinic may further strengthen the basic skills. Finally, experience in standardized reproducible procedures (i.e., bariatric surgery) is necessary to complete the learning process [14].

Several studies have described a changing patient cohort during the learning curve [20,21], and an increasing patient complexity before completing the learning curve was argued to slow down the learning process. In contrast, we could not observe such a trend toward surgically more challenging operations (i.e., T4 tumors or transhiatal extended gastrostomies) during our learning curves. When comparing non-risk-adjusted and risk-adjusted CUSUM, proficiency was not achieved as early, as the risk adjustment was applied. This suggests that the inclusion of more complex cases toward the beginning of the cohort may have prolonged the learning curve due to case selection. However, this is not reflected in the univariate analysis comparing the patient characteristics.

While measuring learning in terms of operating time or length of hospital stay is a reasonable performance indicator for the technique, gaining experience at the expense of patient safety is unacceptable. Thus, the minimum case number for a complete learning curve in terms of mortality and major clinical outcomes must be assured in training programs.

Similarly, the rate of anastomotic leakage was not affected by the level of surgical training. However, a longer OT > 360 was associated with major complications. OT is often considered a surrogate parameter for complexity but is also reported to be independently associated with complications such as surgical site infection (SSI), venous thromboembolism (VTE), bleeding, hematoma formation, and necrosis [20]. These complications are attributable to various time-related factors such as prolonged microbial exposure, diminished efficacy of antimicrobial prophylaxis over time, and increased tissue retraction leading to ischemia. However, reports describing the relationship between an increased OT and postoperative morbidity specifically in LTG are limited.

Our data suggest that consistent oncologic resection was achieved across both groups. In accordance with the German S3-Guidelines, more than 25 lymph nodes were resected in both groups [21]. Similarly, a yield of ≥20 lymph nodes has been reported as sufficient for oncological efficacy in pN3 disease, while more than 10 resected lymph nodes may provide an optimal oncological outcome in pN1 and pN2 disease [22]. In accordance with our results, the value of a lymph node yield over 25 with a low metastatic lymph node ratio (MLNR) may negate the risk of upstaging in postoperative histopathological examination [15].

In addition to perioperative morbidity and mortality, survival (DFS and OS) is one of the most important indicators in curative surgery for gastric malignancy [23]. Since no long-term follow-up assessing the effect of the learning curve on survival has been reported in the literature, this was an additional focus of our study. After a mean follow-up time of 726 and 477 days, respectively, the overall survival and the disease-free survival were comparable in these two phases of learning. However, a limitation of our study was that the shorter follow up time for group B might preclude differences in the overall survival and disease-free survival between groups. We demonstrated the degree of surgical training to be independent of recurrence rates within our observation period. This observation is consistent with previous reports by other investigators [24,25,26]. As our two patient groups had a similar distribution regarding patient characteristics and tumor stage, surgical quality, as measured by comparable and postoperative morbidity and mortality, may have contributed to equal survival curves in our study. Furthermore, successful completion of neoadjuvant and adjuvant chemotherapy occurred at a comparable rate in both groups, indicating a consistent surgical performance that did not impact on LOS.

The limitations of this study include the fact that it evaluated the institutional learning curve, which consists of two surgeon’s patient outcomes collectively rather than evaluating the surgeons individually, as compared to their prior training. On the other hand, both were senior consultants with extensive prior hand-assisted laparoscopic experience, and this fact may underline the necessity of a focused and consistent training to maintain surgical and oncological standards for fellows with less experience. A further limitation was the choice of the primary outcome variable, major complications Clavien Dindo 3–5. Some of these complications (e.g., reoperation for SSI) are less consequential than others. Therefore, a more targeted analysis incorporating only irreversible adverse outcomes should be performed, requiring a larger patient cohort to generate statistical power. Finally, a limitation is that we only used one anastomotic technique. While only one anastomotic technique was employed in this cohort, there has been a dynamic evolution of the anastomosis technique at our institution since this study. The learning curve may have been longer if we avoided using hand-assisting or employed a different technique for esophagojejunostomy. In this case, complexity adjustment could be performed to include this variation in the techniques and assess the effect on the learning curve as well as the perioperative and oncological outcome.

Our study suggests that surgical proficiency in hand-assisted laparoscopic gastrectomy can be achieved with consistent oncological results despite an initial learning curve of 44 cases. The evaluation of proficiency gain in terms of both surgical and oncological outcomes is a crucial component of developing and translating new techniques into clinical practice.

## Figures and Tables

**Table 1:** Patient characteristics and perioperative outcomes.

Characteristic	A, *n* = 45 ^1^	B, *n* = 73 ^1^	*p*-Value ^2^	*q*-Value ^3^
Patient Characteristics				
Sex			0.071	0.18
Male	36 (80.00%)	47 (64.38%)		
Female	9 (20.00%)	26 (35.62%)		
Age (years)	63.2 (12.6)	63.6 (13.6)	0.84	0.84
ASA Score			0.057	0.18
1	4 (8.89%)	3 (4.11%)		
2	15 (33.33%)	37 (50.68%)		
3	24 (53.33%)	33 (45.21%)		
4	2 (4.44%)	0 (0%)		
BMI (kg/m^2^)	26.7 (4.4)	26.0 (4.8)	0.30	0.38
Non-surgical Treatments				
Neoadjuvant chemotherapy	37 (82.22%)	61 (83.56%)	0.85	>0.99
Adjuvant chemotherapy planned	41 (91.11%)	65 (89.04%)	>0.99	>0.99
Adjuvant chemotherapy completed	33 (73.33%)	51 (69.86%)	0.69	>0.99
Localization *				
Antrum	11 (24.44%)	31 (42.47%)	0.047	0.071
Cardia	12 (26.67%)	23 (31.51%)	0.58	0.58
Corpus	26 (57.78%)	27 (36.99%)	0.027	0.071
Operation				
OT (min)	360 (101)	289 (70)	<0.001	<0.001
Number of Retrieved Lymph Nodes (LN Yield)	27.83 (8)	34.51 (12)	<0.001	<0.001
Ratio of affected Lymph Nodes (LN Ratio)	0.122 (0.208)	0.072 (0.136)	0.24	0.37
LOS (Days)	18.0	13.97	0.154	0.234
Total gastrectomy	33 (73.33%)	47 (64.38%)	0.31	0.37
Transhiatal extended gastrectomy	12 (26.67%)	26 (35.62%)	0.31	0.37

^1^*n* (%); mean (SD); ^2^ Pearson’s Chi-squared test; Wilcoxon rank sum test; Fisher’s exact test; ^3^ False discovery rate correction for multiple testing; * Multiple tumor localizations possible. ASA—American Society of Anesthesiologists, BMI—body mass index, OT—operating time, LN—lymph node, LOS—length of hospital stay.

**Table 2:** Histopathological characteristics.

Characteristic	A, *n* = 45 ^1^	B, *n* = 73 ^1^	*p*-Value ^2^	*q*-Value ^3^
Histopathology			0.58	0.45
G1–G3 Adenocarcinoma	43 (95.56%)	68 (93.15%)		
SRCC *	10 (22.22%)	30 (41.10%)		
NET	0 (0.00%)	2 (2.74%)		
MANEC	1 (2.22%)	1 (1.37%)		
Other	1 (2.22%)	2 (2.74%)		
Lauren Classification			0.72	0.83
Intestinal	17 (37.78%)	30 (41.10%)		
Diffuse	23 (51.11%)	38 (52.05%)		
Other	5 (11.11%)	5 (6.85%)		
T3/T4 stage: Preoperative Staging	31 (68.89%)	55 (75.34%)	0.44	0.83
T3/T4 stage: Postoperative Histopathology	24 (53.33%)	35 (47.95%)	0.57	0.83
T-Stage in Preoperative Staging			0.92	>0.99
T0	2 (4.44%)	2 (2.74%)		
T1	3 (6.67%)	5 (6.85%)		
T2	9 (20.00%)	11 (15.07%)		
T3	24 (53.33%)	41 (56.16%)		
T4	7 (15.56%)	14 (19.18%)		
T-Stage in Postoperative Staging			0.61	0.83
T0	3 (6.67%)	7 (9.59%)		
T1	8 (17.78%)	17 (23.29%)		
T2	10 (22.22%)	14 (19.18%)		
T3	19 (42.22%)	32 (43.84%)		
T4	5 (11.11%)	3 (4.11%)		
N (N0/N1/N2/N3)			0.20	0.56
N0	11 (24.44%)	29 (39.73%)		
N1	21 (46.67%)	22 (30.14%)		
N2	7 (15.56%)	15 (20.55%)		
N3	6 (13.33%)	7 (9.59%)		
R1	1 (2.22%)	3 (4.11%)	>0.99	>0.99
L1	13 (28.89%)	11 (15.07%)	0.070	0.45
V1	4 (8.89%)	2 (2.74%)	0.20	0.56
Poor differentiation (G > 2)	25 (55.56%)	38 (52.05%)	0.71	0.83
G			0.64	0.83
G1	1 (2.22%)	3 (4.11%)		
G2	18 (40.00%)	26 (35.62%)		
G3	25 (55.56%)	44 (60.27%)		

^1^*n* (%); ^2^ Fisher’s exact test; Pearson’s Chi-squared test; ^3^ False discovery rate correction for multiple testing; * Sub-classification of G1–G3 adenocarcinoma; SRCC—signet ring cell carcinoma, NET—neuroendocrine tumor, MANEC—mixed adenoneuroendocrine carcinoma.

**Table 3:** Perioperative morbidity and mortality after hand-assisted laparoscopic gastrectomy.

Characteristic	A, *n* = 45 ^1^		B, *n* = 73 ^1^		*p*-Value ^2^
Perioperative Morbidity CD 1–4 (%)	22	48.89%	34	46.58%	0.81
Major Complications, CD 3–4 (%)	12	26.67%	10	13.70%	0.079
Reoperation within 30 days	0	0.00%	2	2.74%	0.52
Surgical Complications					
Anastomotic Leak	3	6.67%	4	5.48%	>0.99
Intraabdominal Abscess	3	6.67%	6	8.22%	>0.99
Acute Diaphragmatic Hernia	2	4.44%	1	1.37%	0.56
Acute Abdominal Wall Dehiscence	0	0.00%	2	2.74%	0.52
SSI	3	6.67%	12	16.44%	0.12
Pulmonary Complications					
Pneumonia	11	24.44%	12	16.44%	0.29
Pleural Effusion	9	20.00%	8	10.96%	0.17
Pneumothorax	3	6.67%	3	4.11%	0.67
Respiratory Failure Requiring Reintubation	2	4.44%	2	2.74%	0.64
Pulmonary Embolus	1	2.22%	3	4.11%	>0.99
Other Complications					
Cardiac Arrest Requiring CPR	0	0.00%	1	1.37%	>0.99
Acute Delirium	3	6.67%	3	4.11%	0.67
Generalized Sepsis	3	6.67%	2	2.74%	0.37
Multiple Organ Dysfunction Syndrome	2	4.44%	1	1.37%	0.56
Urinary Tract Infection	2	4.44%	4	5.48%	>0.99
Gastrointestinal Bleeding	0	0.00%	2	2.74%	0.52
Central Line Infection	2	4.44%	6	8.22%	0.71
30 Day Mortality	0	0.00%	1	1.69%	0.365
90 Day Mortality	1	2.08%	2	3.39%	0.684
1-Year Survival Rate	93.3		96.9		0.976
2-Year Survival Rate	90.6		92.5		0.976

^1^*n* (%); ^2^ Pearson’s Chi-squared test; Fisher’s exact test; CD—Clavien Dindo, SSI—surgical site infection, CPR—cardiopulmonary resuscitation.

**Table 4:** Logistic regression analysis for major complications.

	Univariate		Multivariate	
	HR (95% CI)	*p*-Value *	HR (95% CI)	*p*-Value *
Age > 65	**0.460 (0.189–1.120)**	**0.041**	**5.101 (1.787–14.562)**	**0.002**
Sex (Male)	1.078 (0.398–2.923)	0.882		
ASA ≥ 3 (%)	**2.550 (1.065–6.107)**	**0.036**	2.280 (0.807–6.440)	0.120
SRCC	0.702 (0.220–2.358)	0.587		
Transhiatal extended Gastrectomy	2.332 (0.975–5.578)	0.057		
OT > 240 min	1.721 (0.533–5.552)	0.364		
OT > 360 min	**3.954 (1.578–9.911)**	**0.003**	**4.494 (1.484–13.614)**	**0.008**
Number of retrieved lymph nodes > 25	1.303 (0.439–3.870)	0.634		
Surgical Training	1.037 (0.437–2.462)	0.934		
(Group A: Group B)				
T3/T4	0.912 (0.361–2.300)	0.845		
N > 2	0.854 (0.321–2.272)	0.752		
G > 2	0.774 (0.333–1.799)	0.552		
Vascular invasion	1.574 (0.273–9.074)	0.612		
L	1.076 (0.380–3.045)	0.891		

* Bias-reduced generalized linear regression model. HR—Hazard Ratio, CI- Confidence Interval, ASA—American Society of Anesthesiologists, SRCC—signet ring cell carcinoma, OT—operating time. Significant values are indicated in bold.

**Table 5:** Cox proportional hazard model for overall survival and disease-free survival.

	Disease Free Survival				Overall Survival			
	*p*-Value	Exp (B)	95.0% Confidence Interv. for Exp (B)		*p*-Value	Exp (B)	95.0% Confidence Interv. for Exp (B)	
			Lower	Upper			Lower	Upper
Age	0.734	0.994	0.960	1.029	0.737	0.990	0.937	1.047
Sex (Male)	0.640	1.267	0.470	3.417	0.939	1.071	0.186	6.163
Surgical Training (Group A)	0.370	1.519	0.609	3.789	0.706	1.370	0.267	7.032
SRCC	**0.049**	3.019	1.003	9.088	0.519	1.767	0.313	9.981
G > 2	0.463	1.367	0.593	3.148	0.750	1.257	0.308	5.131
R1	**0.003**	12.815	2.350	69.871	**0.024**	19.103	1.473	247.692
BMI > 30	0.292	0.600	0.232	1.553	0.245	0.281	0.033	2.389
OT > 360 min	0.200	0.564	0.235	1.353	**0.050**	0.234	0.055	1.000

SRCC—signet ring cell carcinoma, G—differentiation, R—completeness of resection, BMI—body mass index, OT—operating time. Significant values are indicated in bold.

## Data Availability

Data are available from the authors upon request.
